# Light Activation of Nanocrystalline Metal Oxides for Gas Sensing: Principles, Achievements, Challenges

**DOI:** 10.3390/nano11040892

**Published:** 2021-03-31

**Authors:** Artem Chizhov, Marina Rumyantseva, Alexander Gaskov

**Affiliations:** Chemistry Department, Moscow State University, 119991 Moscow, Russia; chizhov@inorg.chem.msu.ru (A.C.); gaskov@inorg.chem.msu.ru (A.G.)

**Keywords:** semiconductor gas sensors, light activation, nanocrystalline metal oxides, sensitization, organic dyes, quantum dots, plasmon nanoparticles

## Abstract

The review deals with issues related to the principle of operation of resistive semiconductor gas sensors and the use of light activation instead of thermal heating when detecting gases. Information on the photoelectric and optical properties of nanocrystalline oxides SnO_2_, ZnO, In_2_O_3_, and WO_3_, which are the most widely used sensitive materials for semiconductor gas sensors, is presented. The activation of the gas sensitivity of semiconductor materials by both UV and visible light is considered. When activated by UV light, the typical approaches for creating materials are (i) the use of individual metal oxides, (ii) chemical modification with nanoparticles of noble metals and their oxides, (iii) and the creation of nanocomposite materials based on metal oxides. In the case of visible light activation, the approaches used to enhance the photo- and gas sensitivity of wide-gap metal oxides are (i) doping; (ii) spectral sensitization using dyes, narrow-gap semiconductor particles, and quantum dots; and (iii) addition of plasmon nanoparticles. Next, approaches to the description of the mechanism of the sensor response of semiconductor sensors under the action of light are considered.

## 1. Introduction

The operation principle of semiconductor gas sensors is based on a reversible change in their electrical resistance depending on the composition of the gas phase [1]. This change is due to the interaction of the gas molecules with the surface of the semiconductor sensitive layer, which leads to a change in the concentration of the main charge carriers depending on the content of a particular component of the gas phase. Like any chemical sensor, a semiconductor gas sensor can be characterized by a number of specific parameters, such as the sensor signal value, sensitivity, selectivity, response, and recovery time [2]. The sensor signal *S* can be determined in various ways, for example, as the ratio of the resistance of the sensor in the presence of the target gas to the resistance in the background atmosphere (Equation (1)) or as the ratio of the increase in the resistance of the sensor during gas detection to the resistance of the sensor in the background atmosphere (Equation (2)). The sensor signal is calculated in such a way that its values increase with the concentration of the target gas, so if the resistance of the sensor in the presence of the detected gas decreases, the Formulas (1) and (2) change accordingly:

(1) $$S=\frac{R_{gas}}{R_{air}}\Leftrightarrow S=\frac{R_{air}}{R_{gas}},\;\mathrm{or}\;$$

(2) $$S=\frac{R_{gas}-R_{air}}{R_{air}}\Leftrightarrow S=\frac{R_{air}-R_{gas}}{R_{air}},$$

Traditional materials for gas sensors of the resistive type are wide-gap *n*-type semiconductor metal oxides, such as SnO_2_, ZnO, In_2_O_3_, and WO_3_. This choice is primarily related to their structural and chemical stability, since in the vast majority of cases, for the manifestation of sensor sensitivity of such materials, they need to be heated to a temperature of 200–500 °C. One of the limitations affecting the choice of semiconductor oxide as a material for a gas sensor is its electrical resistivity, which should not be too high, making it difficult to measure (as, for example, in the case of TiO_2_). In order for the processes occurring on the surface of the sensitive layer to have a significant impact on the electrophysical characteristics of the sensor, it is necessary that the material of the sensitive layer has a significant surface. High values of the specific surface area can be achieved in the case of highly dispersed metal oxides, whose crystallite sizes range from units to tens of nanometers. The electrophysical properties of materials based on nanocrystalline metal oxides depend in a complex way on the morphology and grain size, which distinguishes them, for example, from single-crystal semiconductors, whose electrical conductivity is determined only by the concentration and mobility of charge carriers.

At present, considerable attention is paid to the role of electrostatic barriers that arise at the grain boundaries in the formation of the conductive and sensor properties of nanocrystalline metal oxides [3]. Based on this approach, oxygen chemisorption is the main reason for the formation of potential barriers on the surface of semiconductor oxide grains [1]. At low temperatures (near room temperature), the chemisorption of oxygen on *n*-type semiconductor oxides occurs with the capture of the conduction electron and the preservation of its molecular form according to Equation (3):

(3) $$O_{2\left(gas\right)}+e^{-}\to O_{2\left(ads\right)}^{-},$$

At higher temperatures, dissociation of oxygen molecules and its chemisorption in atomic form is possible [4]:

(4) $$O_{2\left(gas\right)}+e^{-}\to 2O_{\left(ads\right)}^{-},\;(>\;180\;{}^{\circ}C)$$

(5) $$O_{\left(ads\right)}^{-}+e^{-}\to O_{\left(ads\right)}^{2-}\;.(400–600\;{}^{\circ}C)$$

To shield the negative charge that occurs on the grain surface, the near-surface region of the crystallites is depleted of electrons that can be considered as an increase in potential barriers at the grain boundaries, leading to a decrease in their electrical conductivity. The average length, which characterizes the depth of the electric field penetration into the semiconductor at small perturbations (under the flat bands condition), is called the Debye length (Equation (6)) [5]:

(6) $$L_{D}=\sqrt{\frac{\varepsilon \varepsilon_{0}k_{B}T}{q^{2}\left(n+p\right)}},$$

where ε_0_ is the permittivity of the vacuum, ε is the static permittivity of the semiconductor, *q* is the value of the elementary charge, *n* and *p* is the concentration of electrons and holes, respectively. The effective width *W_eff_* of the depleted layer with a strong near surface bands curvature is determined by the Equation (7):

(7) $$W_{eff}=L_{D}\sqrt{2\left(Y_{S}-1\right)},$$

here $Y_{S}=qV_{S}/k_{B}T$ is the height of the surface potential of the semiconductor, expressed in dimensionless quantities [5].

Although the processes that cause the appearance of the sensor response of semiconductor oxides may be of different nature, there are only two types of response of a resistive sensor that correspond to an increase or decrease in the electrical conductivity of the sensitive layer relative to its electrical conductivity in the background atmosphere (which, usually, is dry air). In some cases, the dualistic behavior of the sensor response can be associated with the redox properties of the detected gas molecules. For example, *n*-type semiconductor oxides will exhibit the following behavior:

(1) A small group of oxidizing gases, such as *NO*_2_, Cl_2_, and O_3_, cause the electrical conductivity to decrease. This effect is explained by the higher electron affinity of these gases compared to oxygen. As in the case of oxygen, the chemisorption of these gases proceeds with the capture of conduction electrons, leading to even greater depletion of oxide grains by electrons, for example:

(8) $${\mathrm{NO}}_{2\left(gas\right)}+e^{-}\to {\mathrm{NO}}_{2\left(ads\right)}^{-},$$

(2) An increase in electrical conductivity is caused by a larger group of reducing gases (CO, NH_3_, H_2_S, H_2_, CH_4_, etc.) and volatile organic compounds VOCs (formaldehyde, acetone, ethanol, benzene, etc.). It is believed that their interaction with chemisorbed oxygen (Equation (9)) leads to the release of electrons and the reduction of potential barriers at the boundaries of metal oxide grains, for example:

(9) $${\mathrm{CO}}_{\left(gas\right)}+O_{\left(ads\right)}^{-}\to {\mathrm{CO}}_{2\left(gas\right)}+e^{-},$$

The problem of selectivity in this case can be solved by the selection of catalytic additives that promote the oxidation of certain gases in a given temperature range.

(3) A change in the electrical conductivity of semiconductor oxides may result from a change in the oxygen concentration in the gas phase. According to the Equations (3)–(5), an increase in the oxygen concentration in the gas phase leads to an increase in the concentration of the chemisorbed oxygen and a decrease in the electrical conductivity of the sensitive layer, and vice versa.

(4) The influence of humidity is a concomitant factor in the gas detection; however, the magnitude and direction of the effect may be different, depending on the chemical nature of the detected gas and the material of the semiconductor gas sensor.

One of the disadvantages of resistive gas sensors is their high operating temperature, which lies in the range of 200–500 °C. On the one hand, heating is an effective way to activate chemical reactions on the sensor surface, remove the products of these reactions, and increase the electrical conductivity of the sensitive layer. On the other hand, the need for thermal action on the sensitive layer entails a number of undesirable consequences, including:

(1) high power consumption, which limits the use of such sensors in portable and mobile devices;

(2) degradation of the sensor material under prolonged exposure to high temperature due to grain sintering and diffusion processes, which, in the long term, causes the drift of sensor characteristics;

(3) deformation of the sensor layer when the sensor is heated and cooled due to different thermal expansion coefficients of the sensor layer and the substrate;

(4) danger of using the sensor heating element in places and mechanisms where there is a possibility of explosion or fire.

Because of the noted shortcomings, the need to search for sensor materials that can exhibit gas sensitivity without additional heating was revealed. The use of light activation instead of heating, i.e., irradiation of the sensitive layer with UV or visible light, as a gentler and less energy-consuming method of activation, has become widespread [6,7,8,9,10]. 

The purpose of this review is to discuss the current state of light-activated conductometric gas sensors based on metal oxide semiconductors. The main focus is on the analysis of the mechanisms of UV and visible light activation of the gas sensitivity of semiconductor materials, which determine possible approaches to improve their sensor characteristics. In the case of UV activation, the photo- and gas sensitivity of semiconductor metal oxides may be enhanced by chemical modification with noble metal nanoparticles and their oxides, as well as by creating nanocomposites based on various metal oxides. When activated by visible light, the main routes for improving the sensor characteristics of wide gap metal oxides are their doping, spectral sensitization using organic dyes, or particles of narrow-band semiconductors and quantum dots, as well as plasmon nanoparticles of noble metals.

## 2. Photoconductivity of Nanocrystalline Metal Oxides

The use of a fundamentally different activation method caused a revision of the requirements for the sensor materials themselves, as well as the study of the mechanisms of the processes responsible for the sensor response formation. The activation of the gas sensitivity of resistive sensors under illumination is inextricably linked to the appearance of photoconductivity due to the light absorption and the generation of photoexcited charge carriers. The photovoltaic and optical properties of the most common semiconductor oxides used as gas sensor materials are discussed in this section.

Semiconductor oxides used as materials for gas sensors are generally wide-gap (Table 1). The absorption edges of SnO_2_, ZnO, and TiO_2_ due to direct transitions lie in the UV region [11,12,13]. The absorption edge of In_2_O_3_ includes the visible region of the spectrum due to the contribution of indirect (or, according to other data, forbidden) transitions with 2.6 eV to the light absorption [14]. The nature of the absorption of visible light by tungsten (VI) oxide (*E_g_* = 2.6–2.8 eV) is not fully determined and may be a consequence of the contribution of both direct and indirect transitions [15].

These oxides are *n*-type semiconductors and are able to significantly increase their electronic conductivity under the light of a certain range, i.e., exhibit photoconductivity. Thus, if for semiconductor sensors operating under thermal heating, it is necessary to determine the dependence of their electrical conductivity on the composition of the gas phase, then in the case of light activation, the dependence of the photoconductivity on the composition of the gas phase should be determined. Thus, the phenomena of gas sensitivity and photoconductivity are closely related to each other. The increase in photoconductivity is caused by the appearance of nonequilibrium (photoexcited) charge carriers in a semiconductor, and in the case of single-crystal semiconductors is proportional to the change in the concentration of charge carriers (Equation (10)):

(10) $$\Delta \sigma =e\left(\mu_{n}\Delta n+\mu_{p}\Delta p\right),$$

where *σ* is the specific electrical conductivity; *μ_n_* and *μ_p_* are the mobilities of the electrons and holes, respectively; *n* and *p* are the concentrations of the electrons and holes, respectively.

**Figure 1:**
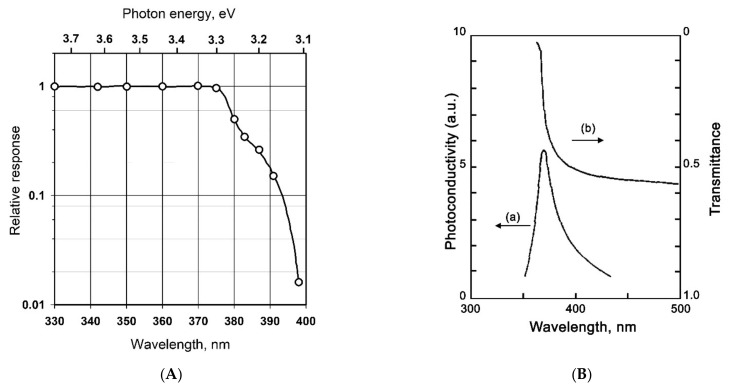
(**A**) Photoconductivity spectrum of monocrystallline ZnO. Adapted from [16], with permission from American Physical Society, 1958. (**B**) Photoconductivity spectrum of monocrystallline SnO_2_ (a) in comparison with its optical absorption spectrum (b) Adapted from [17] with permission from AIP Publishing, 1969.

For semiconductors, there is a correlation between the spectral dependence of the photoconductivity and optical absorption spectra that reflects the causal relationship between the light absorption and the generation of photoexcited charge carriers. Thus, the edge of the increase in the photoconductivity of ZnO single crystals is observed in the range of 3.1–3.3 eV [16] (Figure 1A); the maximum photoconductivity of single-crystal SnO_2_ samples is approximately at 390 nm (Figure 1B), while the position of the absorption edge also correlates with the absorption spectrum of this sample [17].

**Figure 2:**
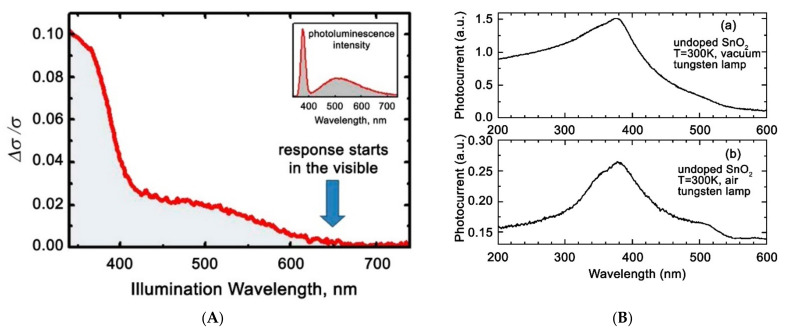
(**A**) Photoconductivity spectrum of ZnO single nanowire. Reprinted from [20] with permission from AIP Publishing, 2014. (**B**) Photoconductivity spectrum of nanocrystallline SnO_2_ in vacuum (a) and in air (b). Reprinted from [21] with permission from Taylor & Francis, 1999.

Nanostructuring of semiconductor oxides significantly affects their photovoltaic properties, including the range of spectral sensitivity. This can be clearly seen in the example of ZnO, which in the single-crystal state practically does not absorb light in the visible range and does not show photosensitivity. At the same time, the literature data indicate the occurrence of photosensitivity of nanocrystalline films and ZnO nanowires in the visible range, starting from 600–650 nm [18,19,20] (Figure 2A). Although the spectral dependence of the photoconductivity of SnO_2_ single crystals also captures a part of the visible spectrum (from 430 nm), polycrystalline SnO_2_ films are characterized by the appearance of a wider range of spectral sensitivity in the visible region (up to 520 nm) (Figure 2B(a,b)) [21]. Data on the spectral dependence of the photoconductivity of In_2_O_3_ and WO_3_ single crystals are not given in the literature, but nanocrystalline samples of these oxides are also characterized by significant photosensitivity in the visible range [22].

**Figure 3:**
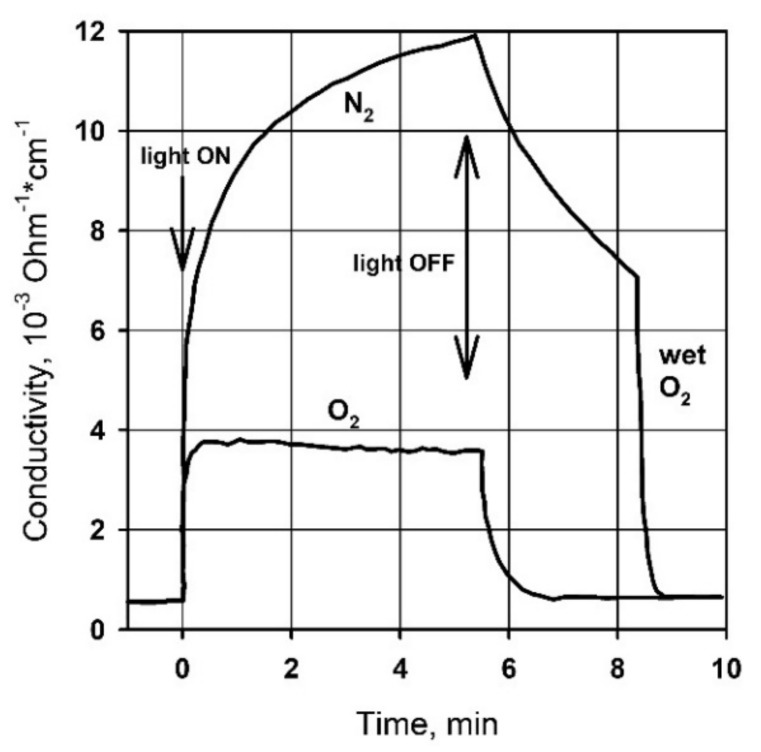
Kinetics of photoconductivity of single-crystal ZnO in an atmosphere of O_2_ and N_2_ under UV radiation. Reprinted from [16] with permission from the American Chemical Society, 1958.

An increase in the dispersion of semiconductor oxides also dramatically slows down the relaxation of photoconductivity. For example, Figure 3 shows that for single-crystal ZnO, the characteristic times of photoconductivity rise and decay in oxygen atmosphere are several seconds. The photoconductivity decay time of nanocrystalline ZnO samples under similar conditions can reach tens of hours [20,23]. This phenomenon, called persistent photoconductivity, is also observed for other nanocrystalline metal oxides [24,25,26].

In general, both the increase in photosensitivity in the visible range and the slowdown in the relaxation of the photoconductivity of nanocrystalline metal oxides can be caused by an increase in the concentration of defects in their crystal structure with a decrease in the crystallites size. It is known that the formation of intrinsic defects in the crystal structure is associated with the occurrence of localized states in the band gap of semiconductors [27]. The excitation of electrons from donor local levels requires less photon energy than for interband transitions, so this process may correspond to an increase in sensitivity in the visible range. The capture of photoexcited electrons at acceptor local levels (traps) is the main reason for the increase in the lifetime of photoexcited charge carriers and the manifestation of persistent photoconductivity.

However, taking into account only internal electronic effects, such as interband transitions and transitions between local levels of intrinsic defects, the photovoltaic properties of semiconductors in real conditions are extremely difficult to interpret. To explain a number of photovoltaic phenomena, the transition from the physical to the physicochemical model of photoconductivity is inevitable. The basis for this physicochemical approach is numerous experimental data demonstrating a significant influence of the composition of the surrounding atmosphere on the value of photoconductivity, its spectral dependence, and relaxation kinetics. For example, Figure 3 shows that the photoconductivity of single-crystal ZnO in an atmosphere of O_2_ and N_2_ under UV radiation is characterized by different relaxation kinetics and the absolute value of the photoconductivity [16]. When measuring in oxygen, the stationary value of the conductivity is reached quite quickly, both when the radiation source is turned on and off. In contrast, in the nitrogen medium, a continuous increase in photoconductivity is observed, while the decrease in photoconductivity when the radiation source is turned off is significantly slowed down compared to the same process in oxygen. The subsequent introduction of wet oxygen into the cell leads to a sharp acceleration of the photoconductivity decay. A similar behavior of photovoltaic properties depending on the composition of the atmosphere was observed for various semiconductor oxides, for example, for a single ZnO nanowire when studying its photoconductivity in vacuum (continuous growth) and in air (rapid achievement of a stationary value, lower photoconductivity value) [19].

The influence of the atmospheric composition on the spectral sensitivity was found, for example, on polycrystalline SnO_2_ films [21]. Although the displacement of the photoconductivity maximum was almost not observed in vacuum and air measurements (in both cases it is observed at about 390 nm), an additional photoconductivity maximum is observed in air measurements at about 510 nm (Figure 2A). The influence of the atmospheric composition on the spectral sensitivity of thin photoconductive films In_2_O_3_ and SnO_2_ was also noted in [28].

Summing up the results of numerous experiments on the effect of atmospheric composition on the photoconductivity of nanocrystalline metal oxides [17,18,20,29,30], it can be concluded that the photoconductivity of semiconductors is significantly affected by the presence of oxygen in the surrounding atmosphere, while in the environment of various inert gases and in vacuum, the photoconductivity behaves in a similar way. This observation leads to the assumption of the possibility of interaction between photoexcited charge carriers and oxygen molecules located on the surface of semiconductor oxides in ion-adsorbed forms.

For example, an increase in the concentration of photogenerated electrons *e_ph_* in the conduction band of a metal oxide can lead to the chemisorption of an additional amount of oxygen on its surface, i.e., to photoadsorption:

(11) $$O_{2\left(gas\right)}+e_{ph}^{-}\to O_{2\left(ads\right)}^{-},$$

The opposite process, photodesorption, is possible when photoexcited holes recombine with electrons captured by chemisorbed oxygen molecules:

(12) $$O_{2\left(ads\right)}^{-}+h_{ph}^{+}\to O_{2\left(gas\right)},$$

In this case, the above mentioned features of changes in the photovoltaic properties of nanocrystalline semiconductor oxides, depending on the composition of the atmosphere, find the following explanation. The continuous increase in photoconductivity in an oxygen-free medium can be a consequence of the continuous photodesorption of oxygen according to Equation (12) and the accumulation of photoexcited charge carriers in the metal oxide grains, leading to a gradual decrease in potential barriers between the grains and an increase in the electrical conductivity of the sample. The decrease in photoconductivity in an oxygen-free medium is mainly due to the recombination of photoexcited charge carriers, which occurs through their capture on defects-traps, and therefore has a delayed character. In an oxygen-containing atmosphere, the rapid achievement of a steady state of photoconductivity is due to the dynamic equilibrium between the rate of generation of photoexcited charge carriers and the rates of oxygen adsorption and photodesorption (Equations (11) and (12)). In this case, the decrease in photoconductivity can be caused not only by the recombination of photoexcited charge carriers through internal defect levels, but also by the regeneration of the surface concentration of chemisorbed oxygen to the initial value, so it has an accelerated character.

Thus, it is essential when considering the photoconductivity of materials based on nanocrystalline oxides to take into account the possibility of interaction of photogenerated charge carriers with molecules adsorbed on the surface of a semiconductor oxide, in particular, with chemisorbed oxygen molecules.

## 3. Activation of Gas Sensitivity of Semiconductor Metal Oxides under UV Light

The study of the influence of the composition of the gas atmosphere on the photovoltaic properties of semiconductors has long been associated with the physical studies, thus expanding the understanding of the processes of generation and recombination of photoexcited charge carriers in these materials. It was only in the early 1990s that pioneering work was carried out to study the effect of light activation on the sensor sensitivity of semiconductor oxides. It was found that the photoconductivity of polycrystalline layers of metal oxides under UV light shows sensitivity to much weaker changes in the composition of the atmosphere than previously demonstrated in physical studies. This allowed the consideration of this activation method on a par with traditional thermal heating.

For example, the authors [31] found a decrease in the photoconductivity of a polycrystalline SnO_2_ film under UV light when acetone and trichloroethylene vapors were introduced into the measuring cell. At a concentration of acetone and trichloroethylene of 760 and 270 ppm, respectively, the relative drop in the electrical conductivity of the SnO_2_ film reached 25–50%. The enhancement of the sensor response of polycrystalline SnO_2_ and In_2_O_3_ films to NO_2_ and CO at room temperature under UV light was demonstrated in the works of the scientific group of Prof. G. Sberveglieri, in 1996–2001 [32,33,34]. In the above-mentioned works, gas-discharge (mercury) lamps were used as a source of UV radiation. Such lamps are usually quite large, of fragile construction, characterized by high power consumption, and require a high voltage source for their operation. Therefore, the study of the effect of UV radiation on the sensor sensitivity of semiconductor oxides was rather fundamental. These studies acquired a pronounced practical orientation with the advent of affordable low-power UV LEDs, which led to the idea of creating compact gas sensors with low power consumption.

The widespread use of low-power UV LEDs to activate the sensor sensitivity of semiconductor oxides has led to increased publication activity in this area since the 2010s. To date, numerous studies have been published on the effect of UV radiation on the activation of the sensor sensitivity of semiconductor oxides, such as SnO_2_ [35,36], In_2_O_3_ [37,38,39,40,41,42,43], ZnO [44,45,46,47,48,49,50,51], WO_3_ [52,53,54,55,56,57,58,59], and TiO_2_ [60,61,62,63,64], of various morphologies. Despite the fact that in some cases the obtained materials demonstrated high sensor sensitivity, many authors noted the low selectivity of materials based on individual binary metal oxides. Attempts to influence the selectivity of UV-activated gas sensors are inherited from thermally activated sensors, and are currently being developed in the direction of chemical modification (sensitization) of the oxide matrix by catalytically active nanoparticles of noble metals, such as Au [65,66,67,68,69], Ag [70,71], Pt [72,73], Pd [74], PdO clusters [75], nanoparticles of transition metals [76], and carbon materials [77,78,79]. For example, it is stated in [72] that modification of SnO_2_ by platinum clusters of 8–10 nm in size leads to a significant (40–70 times) enhancement of the sensor response when detecting hydrocarbon vapors at room temperature and illuminated by a UV radiation source (λ_max_ = 365 nm, 2 μW/cm^2^). It is not uncommon to use several types of catalytic additives to influence the selectivity of UV-activated gas sensors based on semiconductor oxides [80]. In general, this approach can be characterized by the absence of significant differences between the materials used in UV-activated and thermally activated semiconductor gas sensors.

**Figure 4:**
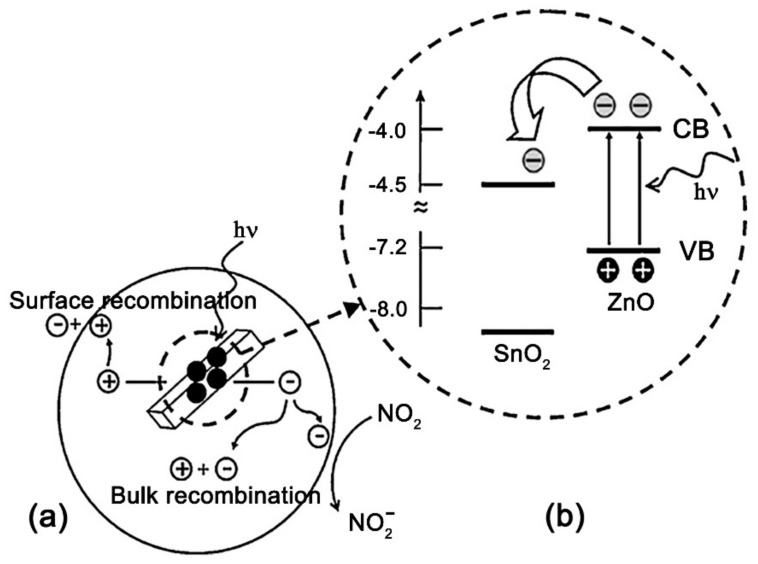
Schematic diagram: (**a**) the carriers transport with UV light stimulated, (**b**) energy band structure and electron–hole pair separation in the ZnO/SnO_2_ heterostructure in the area marked with a dashed circle. Reprinted from [84] with permission from Elsevier, 2012.

As a separate approach to the design of sensor materials for detecting gases under UV illumination, we can distinguish the creation of nanocomposites based on wide-gap semiconductors that form *n-n* heterojunctions among themselves. The gas-sensitive properties of nanomcoposites ZnO/In_2_O_3_ [81,82], ZnO/SnO_2_ [83,84,85,86,87,88], TiO_2_/SnO_2_ [89], Bi_2_O_3_/ZnO [90], and GaN/SnO_2_ [91] have been studied. The creation of such materials is based on the idea of separating photoexcited charge carriers between grains of different semiconductors. This can be illustrated by the energy diagram, which for the ZnO/SnO_2_ system has the following form (Figure 4). Zinc oxide has a smaller band gap (3.2 eV) than tin dioxide (3.6 eV), so it absorbs UV light more effectively, and in this case plays the role of a photocatalyst. Since the Fermi level in ZnO is higher than in SnO_2_, it is possible to transfer photoexcited electrons from ZnO to SnO_2_, and, conversely, photoexcited holes from SnO_2_ to ZnO. The separation of photoexcited charge carriers between different phases increases their lifetime and the probability of interaction with adsorbed gas molecules, in addition, makes this interaction “target” due to the accumulation of different types of charges in different crystal phases. In general, this approach to the creation of materials for UV-activated semiconductor gas sensors is characterized by a variety, both in terms of the components used and in terms of the explanations used to justify the sensor sensitivity of the obtained nanocomposites. It is interesting to note that the idea of creating a sensor material that would combine both of the approaches considered—chemical modification by catalytic particles and the creation of nanocomposites with the function of separating charge carriers—has not yet been implemented.

A short summary of literature data on the sensor signal of wide-gap oxides under UV and visible light activation toward various gases is presented in Table 2.

## 4. Activation of Gas Sensitivity of Semiconductor Metal Oxides under Visible Light

### 4.1. The Role of Impurity Absorption

Further development of the idea to reduce power consumption by replacing thermal activation with light activation led to the understanding that the use of UV radiation to activate the sensor sensitivity of semiconductors does not fully meet the desired requirements for efficiency. LEDs based on InN/GaN/AlN heterostructures, which are most often used as a source of UV radiation, have low efficiency and quantum yield compared to LEDs that emit visible light; the technology of their manufacture remains expensive and imperfect to date [92]. In addition, the molecules of many organic substances, whose detection is of practical importance, as well as NO_2_ molecules [93], are able to undergo destruction under UV light, so the selective and accurate determination of such substances under UV activation can be difficult. From the point of view of the efficiency of converting electrical energy into light using LEDs, it is most appropriate to use the spectral ranges of about 400–450 nm and 600–650 nm, i.e., in the region of blue and red light [94]. In addition, visible light does not lead to the destruction of the molecules of the detected substances. It follows that the use of visible light to activate the sensor sensitivity of semiconductors is a further promising continuation of the idea to create gas sensors with low power consumption and to improve selectivity.

In Section 2, it was already noted that nanocrystalline metal oxides are able to exhibit a photoresponse when exposed to visible light. Similarly, it was found that under visible light, the sensor response of nanocrystalline SnO_2_ [95,96,97], WO_3_ [98,99,100], and ZnO [101,102,103] can also be activated. It should be noted that, speaking about the photoresponse and activation of the sensor properties of wide-gap semiconductor oxides under visible light, two cases should be distinguished:

(1) activation by blue light (in the range ~ 400–500 nm);

(2) activation by green or red light (in the range ~ 500–600 nm).

When activated by light in the blue range, although the wavelength of the exciting radiation formally lies in the visible region, the photon energy is very close to the band gap of metal oxides. There are two main reasons for the photosensitivity of wide-gap nanocrystalline metal oxides when exposed to blue light. First, as is known, in the nanocrystalline state, metal oxides exhibit a significant tailing of the absorption edge, due to the disordering of their crystal structure and the appearance of a significant number of defective (impurity) levels near the edges of the energy bands. As a result, the absorption edge begins to capture the visible part of the spectrum. Secondly, in the case of In_2_O_3_ [104] and WO_3_ [99,100,105], it was shown that their absorption of visible light can be caused by indirect or forbidden transitions, whose energy lies in the range of 2.4–2.6 eV. This allows the listed oxides to demonstrate photo-and gas sensitivity under blue light activation. In both cases, the photoresponse and sensor sensitivity are due to the direct excitation of charge carriers from the valence band to the conduction band, or between states lying near the edges of these bands.

The photoresponse and activation of the sensor properties of wide-gap semiconductor oxides under longer-wavelength radiation (corresponding to the green and red regions of the spectrum) is a more interesting and rarer situation. In this case, the absorption edge of the metal oxide and the emission spectrum of the LED do not overlap. However, these phenomena can also be explained by the photoexcitation of electrons from deeper impurity levels located inside the band gap of these metal oxides. Some authors also associate the photoresponse of wide-gap metal oxides in the long-wavelength spectral region with direct photoionization of chemisorbed oxygen (Equation (13)) [18]:

(13) $$O_{2\left(ads\right)}^{-}\to O_{2\left(gas\right)}+e^{-},$$

In this case, the electrons from the local acceptor levels are excited into the conduction band of the semiconductor oxide, and the electron-neutral oxygen molecule is desorbed. Although this process is not associated with the direct excitation of electrons between the internal energy levels of the semiconductor, it leads to a decrease in potential barriers at the grain boundaries of the semiconductor and the appearance of photoconductivity.

Despite the fact that under visible light, wide-gap semiconductor oxides are able to demonstrate the appearance of a sensor response, this effect is usually quite weak. The main reason for this is the small coefficient of absorption of visible light by these substances. This leads to the fact that only a small part of the emitted photons is absorbed and participates in the formation of electron–hole pairs. To increase the energy efficiency of light-activated sensors, the maximum overlap of the emission spectra of the radiation source and the absorption of the sensitive material is preferred.

There are two fundamentally different ways to create semiconductor gas sensors with visible light activation. One way is to use semiconductors with a smaller band gap, which allows the absorption of visible light photons as the material of the sensitive layer. Such semiconductors are, for example, CdSe [106,107], CdS [108,109,110], MoS_2_ [111], WS_2_ [112], SnS_2_ [113], and BiI_3_ [114], and recently attracting high attention halide perovskite materials [115,116]. Since the use of light activation is aimed at reducing the operating temperature down to room temperature, the sights for the thermal stability of materials under gas detection conditions are lowered, which allows us to consider some halides, sulfides, and other compounds as materials for gas sensors. However, there is a requirement for the photostability of these compounds, which (for example, for sulfides) may not be sufficient in the long term. From this point of view, oxide materials with a small band gap are more promising. Such materials are not yet known very much, but the number of works in this area is rapidly increasing. Among the recent works, we can distinguish the studies of the visible light activated gas sensitivity of V_2_O_5_ [117,118] (*E_g_* = 2.2–2.4 eV) and *p*-type semiconductor oxides, such as HoFeO_3_ [119] and NiO [120]. Another way is to sensitize wide-gap semiconductor oxides to the visible radiation. Among these approaches, one can distinguish doping, spectral sensitization with dyes or particles of narrow-band semiconductors, and sensitization with plasmon metal particles. These approaches will be discussed in more detail in the following sections.

### 4.2. Doping of Wide-Gap Oxides

**Figure 5:**
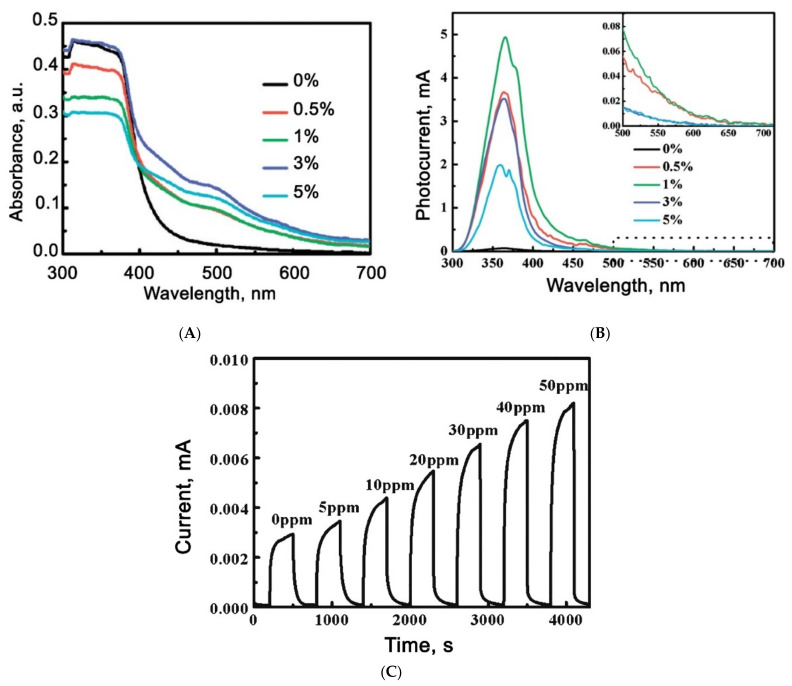
(**A**) Absorbance spectra of pure and Fe-doped ZnO; (**B**) photocurrent spectra of pure and Fe-doped ZnO; gas sensing measurements of 1% Fe-doped ZnO to formaldehyde under green laser illumination; (**C**) Gas sensor measurements to HCHO under 532 nm light illumination. Reprinted from [121] with permission from the American Chemical Society, 2012.

One obvious strategy for enhancing the photosensitivity of wide-gap metal oxides to visible light, which follows from the above examples, is to introduce impurities that contribute to the appearance of an impurity absorption band in the visible range. This strategy has been successfully demonstrated in a number of papers [121,122,123,124,125]. Thus, in [121], the photoelectric and sensor characteristics of ZnO doped with iron in an amount of 0.5–5.0 wt.% were investigated. It can be seen (Figure 5A) that iron doping increases the absorption of ZnO in the visible region. The photosensitivity of ZnO(Fe) increases significantly compared to the pristine ZnO, and also partially shifts to the visible region (Figure 5B). The sensor measurements carried out at room temperature under green light illumination (532 nm, W = 20 mW/cm^2^) showed that the synthesized materials exhibit sensitivity to formaldehyde in the concentration range of 5–100 ppm, while the sensor signal at 100 ppm detection was 287%. The optimal iron concentration, corresponding to the best photoelectric and sensor characteristics, was 1% (Figure 5C).

Despite the comparative simplicity of this approach for increasing the photosensitivity of metal oxide matrices to visible light, its significant disadvantage is the limited solubility of impurities in metal oxides. Therefore, the impurity absorption coefficient is always much smaller than the self-absorption coefficient of light. This does not allow achieving high efficiency when transferring energy from the radiation source to the sensitive layer of the sensor. For example, Figure 5B shows that the photoresponse of iron doped zinc oxide in the visible region is about an order of magnitude smaller than in the UV region corresponding to the intrinsic absorption of ZnO. A significant increase in the impurity concentration eventually leads to the release of a new crystal phase and the formation of a composite material with fundamentally different sensor and optical characteristics.

### 4.3. Spectral Sensitization

Another widespread approach to the sensitization of wide-gap semiconductors to visible light, known from applications in photography [126], photovoltaics [127,128], photocatalysis [129], and other fields [130,131], is the spectral sensitization. This method consists in the immobilization of sensitizer particles on the surface of semiconductor oxides that meet the following conditions:

(1) high absorption coefficient in the visible spectral range;

(2) the ability to transfer the photoexcitation energy to the oxide matrix.

**Figure 6:**
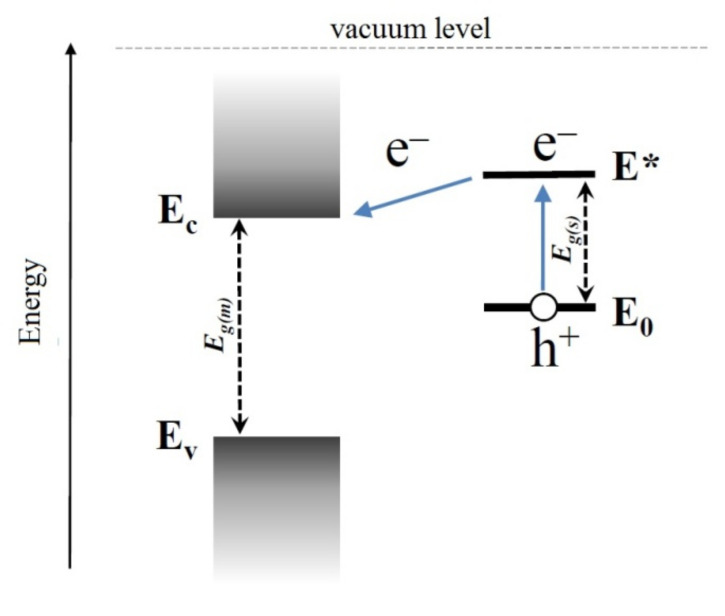
Schematic diagram of photosensitization process.

Organic dye molecules, narrow-gap semiconductor particles, or colloidal quantum dots of semiconductors or metal nanoparticles [132] can act as sensitizers. It is now established that the sensitization effect is caused by the injection of photoexcited charge carriers from the sensitizer particles into the metal oxide matrix. A condition for such a process is the mutual arrangement of the energy levels of the sensitizer and the matrix. For example, spontaneous photoinduced electron transfer from the sensitizer to the metal oxide becomes possible if the position of the excited level of the sensitizer E* is higher in energy than the edge of the conduction band *E_c_* of the semiconductor oxide (Figure 6), while a photoexcited hole remains in the sensitizer particle. A similar scenario is realized with the sensitization of *n*-type semiconductor oxides by organic dyes, CdS, CdSe, PbS, and Ag_2_S nanoparticles. The opposite process—the transfer of photoexcited holes from the sensitizer particles to the oxide matrix—can be realized with a corresponding mutual arrangement of levels, which is realized, for example, for the NiO/CdSe system [133].

The rate constant of the injection of photoexcited electrons is in the range of 1–10 ps, and in the framework of the Marcus theory [134] is approximately proportional ${\left(E^{*}-E_{c}\right)}^{1/2}$. A more precise theory [135] considers the total change in free energy, which takes into account the energy spent on the polarization of the sensitizer particles and the energy spent on the separation of the photoexcited electron–hole pair. Since the characteristic injection times of photoexcited electrons are several orders of magnitude shorter than the lifetime of the excited state under photoluminescence (1–10 ns), the latter process is suppressed if it is possible to inject photoexcited electrons. A competing process for electron injection is the nonradiative recombination process associated with the capture of photoexcited electrons on traps near the bottom of the conduction band, since it has close characteristic times.

#### 4.3.1. Dyes Sensitization

**Figure 7:**
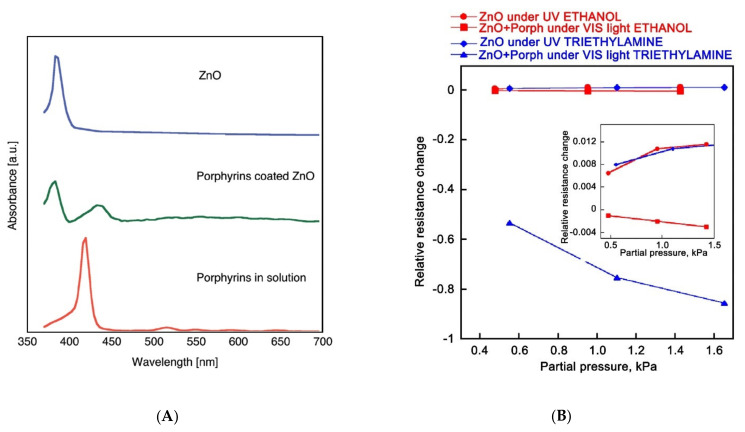
(**A**) Absorbance spectra of pure ZnO, porhirin-coated ZnO, and porphyrins in solution. (**B**) Resistive change at gas sensing measurements of pure and porphyrins-coated ZnO to ethanol and thimethylamine under light illumination. Adopted from [136] with permission from the American Chemical Society, 2012.

Dyes sensitization of gas-sensitive materials is a fairly common approach. A series of studies on the sensor properties of ZnO nanorods sensitized with porphyrin derivatives has been published in [136,137,138,139]. For example, in [136], a methanol solution of 5-(4′-carboxyphenyl)-10,15,20-triphenylporphyrin was deposited on a layer of ZnO nanorods grown on the surface of ITO, after which the resulting structure was dried in air at room temperature. The absorption spectrum of the sensitizer has a maximum at 418 nm; however, when applied to ZnO, it undergoes a red shift. ZnO sensitized with a porphyrin derivative has a maximum absorption in the visible region at 440 nm (Figure 7A). A significant (five-fold) increase in the dark conductivity of the structure was observed compared to the unsensitized layer of ZnO nanorods. Sensor properties were studied at room temperature under the UV and visible light towards ethanol and triethylamine vapors of high concentration (about 0.5–1.5 vol%). The authors of the work were able to establish that under these conditions, the sensor is sensitive to triethylamine vapors, while the presence of ethanol vapors does not affect the electrical conductivity of the sensor (Figure 7B).

In other studies, heterocyclic ruthenium complexes, known from applications in solar energy as effectively light-absorbing dyes, were used to sensitize wide-gap metal oxides. The sensor sensitivity of such hybrid materials to changes in the concentration of oxygen, carbon monoxide [140], and nitrogen dioxide [141,142] at room temperature under visible radiation sources was established. Among the dyes that have been used to sensitize metal oxide sensors, tetratiafulvalene [143], perilenediimide [144], and copper phthalocyanine [145] can also be noted.

The difficulty of using dyes as photosensitizers is associated with the tendency to various aggregations of their molecules, while the optical properties of the aggregates differ significantly from the properties of the original (non-aggregated) dye. This makes it difficult to predict the optical properties of sensitized materials. The sufficient stability of organic dyes under conditions of long-term gas detection under illumination is also questionable, since the authors usually do not provide data on the long-term stability of the sensor properties of the obtained hybrid structures.

#### 4.3.2. Sensitization by Particles of Narrow-Gap Semiconductors

In the literature, there is information about the sensitization of wide-gap metal oxides by Ag_2_S [146], CuO [147], CdS [148,149,150,151,152,153], CdSe [154], SnS_2_ [155], and CH_3_NH_3_SnI_3_ [156] nanoparticles. In fact, this approach is close to one discussed in Section 3 for creating gas-sensitive nanocomposites based on metal oxides, with the difference that the particles responsible for light absorption are semiconductors with an even smaller band gap that allows them to absorb visible light photons. In most studies, the formation of narrow-gap semiconductor particles occurs in situ on the surface of the oxide matrix, which makes it possible to achieve a heterojunction that facilitates the injection of photoexcited electrons. As a result the nanocomposites demonstrate high photosensitivity to visible light. The ratio of light current to the dark one for such nanocomposites can reach 10–100.

**Figure 8:**
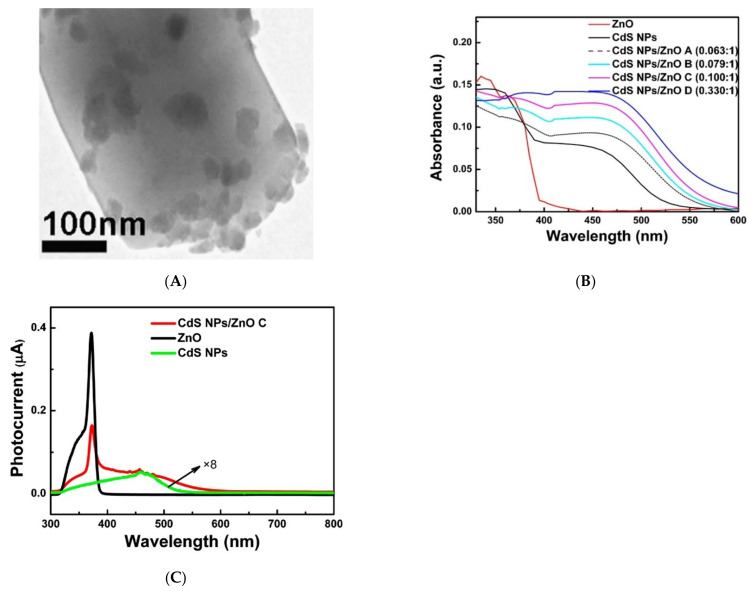
(**A**) Electron microscopy image of ZnO/CdS nanocomposite; (**B**) Absorbance spectra of ZnO, CdS nanoparticles, and ZnO/CdS nanocomposites; (**C**) photocurrent spectra of ZnO, CdS nanoparticles, and ZnO/CdS nanocomposites. Reprinted from [148] with permission from Elsevier, 2010.

As an example, we can cite the work [148], in which the ZnO/CdS nanocomposites were obtained from commercial ZnO and a solution of CdCl_2_ and thiourea under the powerful ultrasonic action. After separating the excess CdS particles, the nanocomposites containing from 5.9% to 24.8% CdS were obtained. According to electron microscopy data, CdS particles with an average size of 10–30 nm were formed on the surface of ZnO grains (Figure 8A). As in the case of dye sensitization, the absorption spectrum of zinc oxide sensitized by CdS nanoparticles shows a significant increase in absorption in the visible region, in this case in the range of 400–600 nm (Figure 8B). The spectral dependence of the photocurrent of the ZnO/CdS structure also shows an increase in the visible region of the spectrum (Figure 8C). The sensor properties of the ZnO/CdS nanocomposites were studied at room temperature under the radiation of a powerful xenon lamp equipped with a filter that cuts off radiation with a wavelength of < 450 nm. The authors of the work noted the appearance of a sensor response when formaldehyde was introduced into the measuring cell in the concentration range of 110–660 ppm.

The disadvantages of these studies include the lack of the data on the concentration dependence of the photoelectric and sensor properties on the content of photosensitizer particles in the nanocomposites. It is interesting to note that the results presented in [146,148] show that the photo- and sensor response of both individual narrow-gap semiconductors and wide-gap metal oxides under visible light is much smaller than for nanocomposites based on the same narrow-gap semiconductors and metal oxides. This effect can be explained by the increased lifetime of the photoexcited charge carriers due to their separation between different phases. Based on this, it can be assumed that the sensor sensitivity of such semiconductor materials is determined not so much by the ability to absorb light, but rather by the existence of suitable conditions for the generation and effective separation of photoexcited charge carriers.

#### 4.3.3. Sensitization with Semiconductor Quantum Dots

A special case of sensitization with the narrow-gap semiconductor nanoparticles is sensitization with colloidal quantum dots (QDs), which are isolated nanocrystals of semiconductors with dimensions less than the Bohr exciton radius in this material. The surface of colloidal QDs is stabilized by ligands preventing their aggregation. The use of semiconductor QDs to modify the optical properties of semiconductor oxides has a number of advantages:

(1) Due to the effect of quantum limitation, the optical properties of QDs made of the same material can be varied widely, changing only their size, as in the case of, for example, cadmium chalcogenides [157].

(2) Due to the fact that colloidal QDs are synthesized and isolated in a separate chemical process, their structure, size, composition, and morphology can be independently and accurately controlled. For example, existing synthesis methods allow the synthesis of CdSe QDs with a relative radius deviation of no more than 5% [158].

(3) Colloidal synthesis methods allow us to create core/shell structures based on quantum dots, with a controlled composition, core size, and shell thickness [159]. The use of such heterostructures has the advantages due to the effects of the initial separation of photoexcited charge carriers inside the photosensitizer [160].

(4) Unlike dyes, whose adsorption on the surface of semiconductor oxides is often accompanied by aggregation and changes in optical characteristics, semiconductor quantum dots, as a rule, retain their inherent optical properties, which allows more accurate predicting and regulating the optical properties of the nanocomposite as a whole.

**Figure 9:**
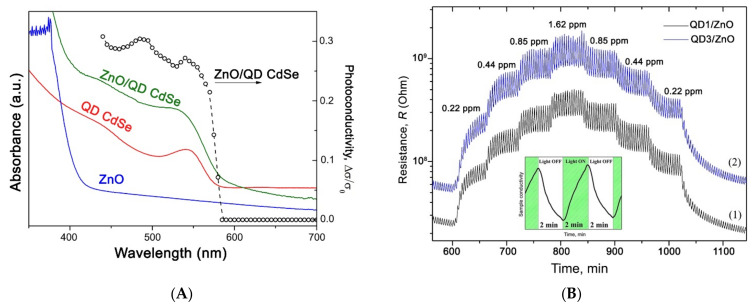
(**A**) Absorbance spectra of ZnO, CdSe QDs, and ZnO/QD_CdSe nanocomposite; (**B**) Gas sensor measurements of ZnO/QD_CdSe nanocomposites to NO_2_ under periodic green light illumination and room temperature. Reprinted from [161] with permission from Esevier, 2014.

The use of CdSe [160,161,162,163,164,165] and PbS [166] quantum dots for the creation of photo- and gas sensitive nanocomposites is described in the literature. The sensor and photoelectric properties of nanocomposites based on semiconductor metal oxides ZnO, SnO_2_, In_2_O_3_, and CdSe colloidal QDswere studied in [161,162,163,164]. For sensitization, spherical CdSe quantum dots with an average diameter of 2.7 nm, stabilized with oleic acid, were used. Such QDs have an absorption maximum at 535 nm, corresponding to the first exciton transition, and are characterized by photoluminescence in the visible range. The spectral dependence of the photoconductivity of the ZnO/QD_CdSe nanocomposite shows a maximum, which position corresponds to the maximum absorption of CdSe QDs (Figure 9A). The ZnO/QD_CdSe nanocomposite demonstrated sensor sensitivity towards NO_2_ at room temperature, under green light (LED, λ_max_ = 535 nm, 20 mW/cm^2^) (Figure 9B). Under similar conditions the nanocomposites based on nanocrystalline SnO_2_, In_2_O_3_, and CdSe QDs also demonstrated enhanced photosensitivity and sensor sensitivity to NO_2_ compared to individual nanocrystalline metal oxides. Gas sensitivity to NO_2_ under blue light irradiation (λ_max_=470 nm, 8 mW/cm^2^) of a nanocomposite based on nanocrystalline ZnO and cubic-shaped perovskite CsPbBr_3_ colloidal quantum dots is demonstrated in the work [167].

### 4.4. Using the Plasmon Resonance Effect

Separately, we should consider the sensitization of semiconductor oxides by nanoparticles that exhibit the effect of plasmon resonance, since in this case the mechanism of sensitization is more complex. Plasmon resonance is understood as the phenomenon of collective oscillation of valence electrons relative to the atomic core, which coincides in frequency with external radiation. An example is gold nanoparticles, for which the plasmon resonance effect is observed in the range of 500–540 nm. The undoubted advantage of noble metal particles is their stability and high light extinction coefficients. This makes their inclusion in the composition of photosensitive materials for light activated gas sensors very promising. In this case, metal nanoparticles are primarily components that ensure the specificity of light absorption in the required spectral range. The nanocomposites based on semiconductor oxides and noble metals were already mentioned in Section 3; however, the role of the latter was in chemical modification, which made it possible to achieve selectivity in interaction with the detected gases. 

In [168], several effects are identified that cause sensitization by plasmon nanoparticles.

(1) The main effect, apparently, is related to the fact that near the particle with the plasmon resonance, an inhomogeneous electromagnetic field is formed, reaching a high intensity at certain points. In a semiconductor, the rate constant of electron–hole pair formation is proportional to the electric field strength, so the rate of generation of electron–hole pairs increases in metal oxide grains that are in the field of a plasmon nanoparticle. It should be noted that this effect is likely not lead to a shift in the photosensitivity of the material in the visible region, but enhances the photosensitivity of the original semiconductor oxide in the UV region, while the effects of radiation in the visible range required for the manifestation of the effect of plasmon resonance.

(2) The sensitizing effect can be caused by direct injection of photoexcited “hot” electrons from plasmon nanoparticles into the conduction band of the semiconductor oxide. The injection of photoexcited electrons can be detected by increasing the photoconductivity of the nanocomposite material when irradiated with light corresponding to the absorption band of plasmon nanoparticles. However, in most studies, the photoconductivity of nanocrystalline oxides sensitized by plasmon nanoparticles increases slightly under such conditions. 

(3) The effective scattering of resonant photons between plasmon particles has an additional effect. As a result, the optical path is lengthened, and the probability of light absorption by the semiconductor matrix increases.

(4) When plasmon nanoparticles absorb light, local heat is released.

**Figure 10:**
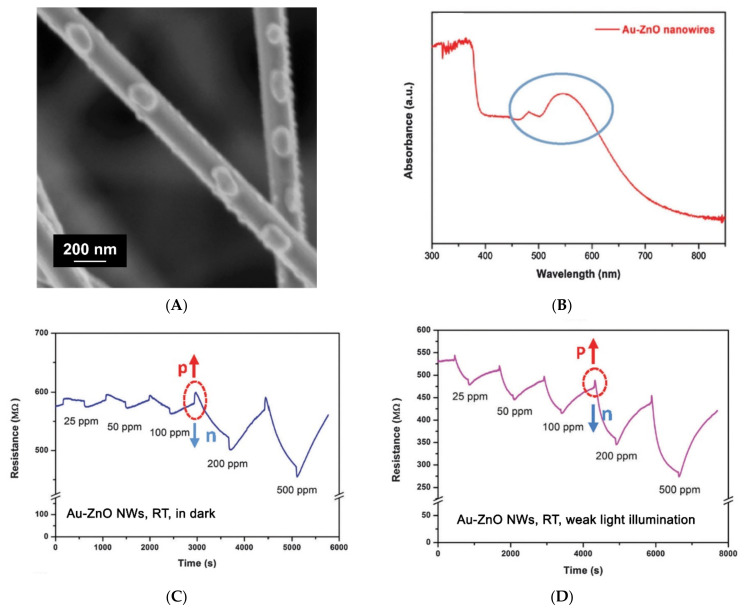
Electron microscopy image (**A**) and absoorbance spectrum (**B**) of ZnO/Au nanocomposite. Gas sensor measurements of ZnO/Au nanocomposite to acetylene in dark (**C**) and under 532 nm light illumination (**D**) and room temperature. Reprinted from [169] with permission from Royal Society of Chemistry, 2015.

A relatively small number of reports have been published on the visible light activated gas sensitive properties of nanocomposites containing plasmon metal nanoparticles [169,170,171,172,173,174,175,176]. As an example, we can cite the work [169], in which the sensor properties of the ZnO/Au nanocomposite to C_2_H_2_ were investigated. The nanocomposite obtained in this work consisted of ZnO nanowires with a thickness of about 100 nm, covered with Au island particles with a size of about 80 nm (Figure 10A). The absorption spectrum of the ZnO/Au nanocomposite contains a wide peak in the visible region with a maximum at about 550 nm, due to the presence of Au nanoparticles (Figure 10B). At room temperature, under green light (λ_max_ = 532 nm), a decrease in the electrical resistance of the ZnO/Au nanocomposite from 580 to 530 MOhm, as well as an increase in the sensor response to 100 ppm C_2_H_2_ by about 10 times were observed (Figure 10C,D). Unfortunately, the results of sensor measurements for individual ZnO nanowires under similar conditions are not presented, which does not allow us to reliably judge the role of gold nanoparticles in the formation of the photo- and sensor response of the nanocomposite. The level of the observed photosensitivity of the nanocomposite is comparable to the photosensitivity of nanocrystalline ZnO under visible light illumination. 

Often, the selected experimental conditions do not allow us to identify the effects achieved by introducing plasmon nanoparticles into the oxide matrix. Thus, the authors [170] showed a 2–3-fold increase in the sensor response of the ZnO/Au nanocomposite on porous silicon to 50–500 ppm NH_3_ at room temperature; however, a continuous radiation source with a spectrum close to solar ( λ > 400 nm) and a sufficiently high intensity (60 mW/cm^2^) was used for illumination. The authors did not provide data on the sensor sensitivity of the structure without Au plasmon nanoparticles under similar conditions. Therefore, it is possible that the observed photosensitivity of the ZnO/Au nanocomposite may be a consequence of the intrinsic photosensitivity of zinc oxide to visible light.

The role of Au plasmon nanoparticles in the formation of the sensor response is more clearly demonstrated in [172], where the data of sensor measurements under illumination are provided for both the ZnO/Au nanocomposite structure and for individual ZnO nanotetrapods. According to the data obtained, the ZnO/Au nanocomposite exhibits selectivity to ethanol vapors under illumination at room temperature, and the presence of Au plasmon nanoparticles enhances the sensor response by 10–12 times. However, a mercury lamp emitting both in the visible and UV light was used for illumination, which casts doubt on the authors’ claim that the mechanism of enhancing the photo-and sensor response of the ZnO/Au nanocomposite is associated with the injection of “hot” electrons from the Au plasmon nanoparticles. Another possible mechanism is the increase in the rate constant of the formation of electron–hole pairs in ZnO during UV light absorption under the action of an electric field that appears due to the plasmon resonance during the visible light absorption by Au particles.

The literature data on the sensor signal of sensitized wide-gap oxides under visible light activation toward various gases are summarized in Table 3.

## 5. Mechanisms of Light Activated Gas Sensing

The interaction of the sensitive material with radiation leads to a complex of various phenomena. The most general approach explains the appearance of the light activated sensor sensitivity of semiconductors based of two types of phenomena: (i) the generation of photoexcited charge carriers and (ii) their interaction with adsorbed gas molecules. The first process leads to an increase in the concentration of free charge carriers, and as a result, an increase in the electrical conductivity of the sensitive semiconductor layer. The second process is caused by the capture and recombination of photoexcited charge carriers with chemisorbed gas molecules that lead to a change in their concentration and, consequently, the steady-state photoconductivity value. The question about the mechanism of sensor response is to determine the specific means of generation, transport of photoexcited charge carriers, and their interaction with chemisorbed gas molecules.

The list of gases that can be detected by light activated semiconductor sensors is quite wide [177]. First of all, it should be noted that the nature of the response of semiconductor sensors under light and thermal activation, as a rule, coincides, that is, in both cases, oxidizing gases lead to a decrease in the electrical conductivity of the sensitive layer, and reducing gases cause its increase. Thus, it can be assumed that the mechanisms responsible for the formation of the sensor response, both during thermal and light activation, should have common features.

The study of the mechanism of sensor sensitivity based only on data on photoconductivity and its changes depending on the composition of the gas atmosphere is not very informative. The electrical conductivity of the sensitive layer is an integral characteristic, and in the case of nanocrystalline semiconductor oxides, its dependence on the concentration of charge carriers, their mobility, and the presence of adsorbed molecules on the surface of the oxide grains is quite complex. The application of the barrier conductivity model to real nanocrystalline materials is also associated with a number of difficulties, for example, in calculating the Debye length and the specific height of potential barriers at the grain boundary. In addition, there are other models of conductivity (for example, hopping, percolation), the use of which can also be justified in the case of nanocrystalline metal oxides, due to their high defectiveness and disorderliness (which mainly concerns the atoms at the grain boundaries).

As a result, when studying the mechanisms responsible for the formation of light activated sensor response, it is necessary to involve additional methods that allow, for example, to identify reacting particles on the surface of the sensitive layer in an independent way. Examples include in situ IR spectroscopy and in situ mass spectrometry. However, such specific methods, although they provide unique information about the transformations of the detected gas molecules on the surface of semiconductors, are still very limited in their use in research on this topic. The importance of using additional research methods in studying the mechanisms of photostimulated processes can be illustrated by the example of the “oxygen-ZnO” system. From numerous experimental data, it is known that the conductivity of zinc oxide, as well as ZnO based nanostructures, in an oxygen-containing atmosphere, increases under UV illumination, although there is a tendency to conductivity decrease with increasing oxygen concentration in the gas phase. The increase in photoconductivity in this case is often associated with the process of photodesorption of oxygen from the ZnO surface (Equation (12)), which leads to a decrease in potential barriers between ZnO grains. However, manometric [178] and mass spectrometric [179] studies have shown that both photodesorption and photoadsorption of oxygen can be observed on the ZnO surface under UV radiation, depending on the synthesis method and stoichiometry of the samples, which is rarely noticed when discussing the mechanism of formation of the sensor response. The specifics of the photostimulated interaction of such semiconductor oxides as SnO_2_, In_2_O_3_, and WO_3_ with oxygen have not been studied by either manometric or mass spectrometric methods.

### 5.1. Light-Activated Sensor Response to Oxidizing Gases

The largest number of works, apparently, is devoted to the study of the photoactivated sensor response of semiconductor oxides in the detection of nitrogen dioxide, which, to some extent, has become a model gas for such investigations. The reason for this is the high sensitivity to NO_2_ (in a number of works declared at the level of tens of ppb), good reproducibility, and a high sensor response. Almost all metal oxides studied demonstrate high sensitivity to NO_2_, even without the need for any special modification of their composition, morphology, stoichiometry, or the size of nanocrystals. Although the mechanism of the sensor sensitivity of semiconductor oxides to NO_2_ is also widely covered in the literature, none of the proposed mechanisms are generally accepted to date.

In general, we can distinguish two processes responsible for the appearance of NO_2_ sensor response of *n*-type semiconductor oxides: (i) NO_2_ adsorption on the surface of semiconductor oxides, leading to a decrease in conductivity; (ii) desorption of NO_2_ from the surface of semiconductor oxides, ensuring a return of conductivity to the base level. The first process at room temperature proceeds spontaneously, the second one requires energy consumption through thermal or light activation.

The adsorption of NO_2_ from the gas phase, which proceeds with the capture of conduction electrons, can be written as Equation (8). Since the electron affinity of the NO_2_ molecule is significantly higher (2.27 eV) than that of the O_2_ molecule (0.45 eV), a possible process is also the displacement of some of the chemisorbed oxygen species from the surface of the semiconductor oxide (Equation (14)):

(14) $${\mathrm{NO}}_{2\left(gas\right)}+O_{2\left(ads\right)}^{-}\to {\mathrm{NO}}_{2\left(ads\right)}^{-}+O_{2\left(gas\right)},$$

There is also a point of view [84] that the chemisorption of NO_2_ is accompanied by its dissociation into NO and O_2_; thus, an additional amount of chemisorbed oxygen appears on the surface:

(15) $$2{\mathrm{NO}}_{2\left(gas\right)}+e^{-}\to 2{\mathrm{NO}}_{\left(gas\right)}+O_{2\left(ads\right)}^{-},$$

Other works suggest more complex processes describing the transformations of NO_2_ molecules during chemisorption on semiconductor oxides [43,81]:

(16) $$2{\mathrm{NO}}_{2\left(gas\right)}+O_{2\left(ads\right)}^{-}+2e^{-}\to {\mathrm{NO}}_{2\left(ads\right)}^{-}+2O_{\left(ads\right)}^{-},$$

(17) $$2{\mathrm{NO}}_{2\left(gas\right)}+O_{2\left(ads\right)}^{-}+2e^{-}\to 2{\mathrm{NO}}_{\left(gas\right)}+2O_{\left(ads\right)}^{2-}.,$$

All the considered processes lead to the accumulation of a negative charge on the surface of metal oxide grains, which leads to an increase in the width of the depleted layer and a decrease in electrical conductivity, and correlates with the results of sensor measurements. Unfortunately, the proposed mechanisms (Equations (14)–(17)) are speculative and have not been supported by additional research.

**Figure 11:**
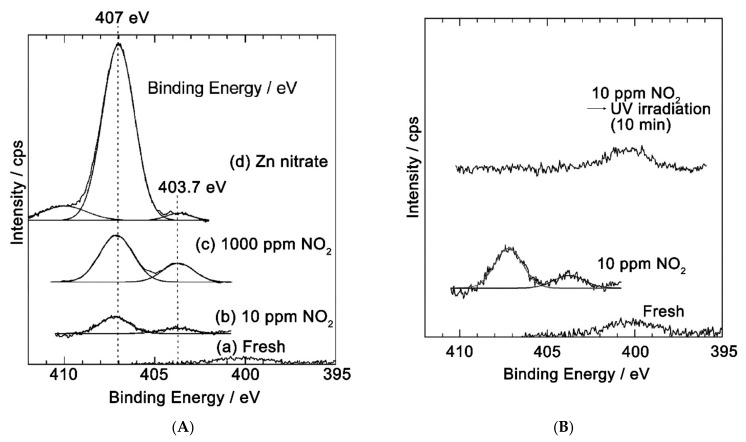
(**A**) XP-spectra on the N1s region for initial ZnO and exposed with 10 and 1000 ppm NO_2_; (**B**) XP-spectra on the N1s region for ZnO exposed with 10 ppm NO_2_ and after UV illumination. Reprinted from [181].

Turning to the study of NO_2_ interaction of with the surface of semiconductor oxides by independent methods, it is necessary to note the works in which the methods of XANES and XPS were used. Thus, in [180], the XANES and XPS methods showed that at room temperature, NO_2_ chemisorption on the ZnO surface occurs mainly with the formation of nitrate anions; however, the results obtained characterize this process at a relatively high (450–500 Torr) NO_2_ pressure, which is significantly different from the operating range of gas sensors. In [181], the chemisorption of NO_2_ was investigated by XPS in the concentration range closer to the operating ranges of gas sensors, namely, when ZnO thin films were exposed to air containing 1000 and 10 ppm of NO_2_. For both NO_2_ concentrations, on the ZnO surface, two nitrogen charge states are observed, corresponding to the NO_3_^−^ (407 eV) and NO_2_^−^ (403.7 eV) ions (Figure 11A). In a sample exposed at 10 ppm NO_2_, the N1s photoelectron lines resulting from NO_2_ adsorption completely disappear when exposed to UV radiation (Figure 11B). It follows from the obtained data that when the ZnO surface is exposed to small (about 10 ppm) concentrations of NO_2_, nitrogen dioxide is actually chemisorbed to form NO_2−_ and NO_3_^−^ ions, and under UV radiation the resulting nitrogen-containing ions are completely photodesorbed. Unfortunately, the O1s spectra of oxygen are not given in this paper, which does not allow us to judge the possible relationship between the processes of NO_2_ and O_2_ sorption. Thus, in addition to Equation (8), the adsorption mechanism should include the possibility of the formation of nitrate ions on the surface of ZnO, by, for example, the reversible interaction of chemisorbed NO_2_ molecules with the lattice oxygen of ZnO (Equation (18)):

(18) $${\mathrm{NO}}_{2\left(gas\right)}+O_{lat}+e^{-}\to {\mathrm{NO}}_{3\left(ads\right)}^{-},$$

Desorption of chemisorbed NO_2_ species, which is necessary to ensure the reversible operation of the sensor, can occur under UV light due to the recombination of electrons captured by NO_2_ molecules with photoexcited holes (Equation (19), similar to the previously considered mechanism of O_2_ photodesorption, Equation (12)):

(19) $${\mathrm{NO}}_{2\left(ads\right)}^{-}+h_{ph}^{+}\to {\mathrm{NO}}_{2\left(gas\right)},$$

Although it was clearly shown in [181] that nitrogen dioxide is desorbed from the ZnO surface under UV radiation, it remains unclear, for example, whether the desorption of unchanged NO_2_ molecules occurs or is accompanied by dissociation into NO and O.

### 5.2. Light-Activated Sensor Response to Reducing Gases

Most authors believe that the response to reducing gases is due to their reaction with chemisorbed oxygen, which leads to a decrease in its surface concentration and an increase in the concentration of conduction electrons in the semiconductor oxide. Thus, the occurrence of sensor sensitivity is explained in a similar way as in the case of thermal activation. For example, when detecting formaldehyde at room temperature, this reaction can be written as Equation (20):

(20) $${\mathrm{HCHO}}_{\left(ads\right)}+O_{2\left(ads\right)}^{-}\to {\mathrm{CO}}_{2\left(ads\right)}+H_{2}O_{\left(ads\right)}+e^{-},$$

It is debatable why this reaction does not occur at room temperature under dark conditions, but occurs under UV or visible light. According to a common point of view, the oxidation reactions of type (20) involve photo-adsorbed oxygen molecules, which are more loosely bound to the surface of the semiconductor oxide than “ordinary” chemisorbed oxygen molecules, and have a greater reactivity [46,69]. At high temperatures, in accordance with Equations (4) and (5), the photoactivated reactions should be considered with the corresponding charge forms of oxygen (O^−^ or O^2−^).

Similarly, the authors [182] justify the sensor sensitivity of polycrystalline ZnO to hydrogen, emphasizing that the photoadsorbed molecules are weakly bound to the surface and can be easily removed from it when the backlight is turned off. However, this statement contradicts the mass spectrometry data, indicating that the photoadsorption of oxygen on the ZnO surface is irreversible [179]. In general, the literature does not yet contain any convincing evidence of the occurrence of reactions of the type (20) on the surface of semiconductor oxides under light activation, obtained, for example, by isotopic mass spectrometric methods, so the proposed mechanism can be considered speculative.

At the same time, the authors [44,54,66,71,183,184] suggested that when volatile organic compounds (acetone, acetaldehyde, ethanol, and hydrocarbons) are detected under UV radiation, photolysis of analyte molecules occurs on the surface of the semiconductor oxide. This facilitates their subsequent oxidation with chemisorbed oxygen, which leads to a change in the conductivity of the semiconductor. TiO_2_ and ZnO are most active in photocatalytic oxidation. Varying the wavelength of UV radiation allows one to change the sensitivity to different gases, providing an increase in the selectivity of the sensor [44].

When explaining the mechanism of sensor properties, some authors consider the direct interaction of chemisorbed molecules of organic substances with photoexcited charge carriers, similar to photocatalytic reactions [49,185,186,187]. For example, in work [49], oxidation of ethanol vapors on the ZnO surface under UV irradiation is described by the following processes (Equations (21)–(23)):

(21) $$C_{2}H_{5}{\mathrm{OH}}_{\left(ads\right)}+\;h^{+}\;\to {\mathrm{CH}}_{3}{\mathrm{CHO}}_{\left(ads\right)}+\;H^{+},$$

(22) $${\mathrm{CH}}_{3}{\mathrm{CHO}}_{\left(ads\right)}+\;2O_{2\left(ads\right)}+h^{+}\;\to 2{\mathrm{CO}}_{2\left(ads\right)}+H_{2}O_{\left(ads\right)}+\;2H^{+},$$

(23) $$2H^{+}+\;2e^{-}\to H_{2\left(ads\right)},$$

Due to both photoexcited holes and photoexcited electrons are involved in the process of photochemical oxidation, an increase in the conductivity of the sensor occurs and a sensor signal appears. Likewise, reinforcing their reasoning with IR spectroscopy, the authors of the work [185] explain the gas sensitivity of TiO_2_ under UV light to CO, but not to H_2_. During the proposed processes (Equations (24) and (25)), adsorbed CO offered electrons to TiO_2_, which led to an increase in surface conductivity TiO_2_ in accordance with the electrochemical reactions on its surface:

(24) $$\mathrm{CO}+H_{2}O\;\to {\mathrm{CO}}_{2}+{\;H}^{+}+\;2e^{-},$$

(25) 12O2+2H++2e−→H2O,

A common feature of the studies concerning the gas sensitivity of semiconductor oxides to reducing gases and volatile organic compounds under light activation is a low sensor response. Typical values of the sensor signal usually do not exceed a few units, and often even a few percent. At the same time, when detecting NO_2_ in a similar concentration range, the sensor signal can reach 10^2^ –10^3^. When interpreting low values of the sensor signal, it should be taken into account that a comparable response can be observed when the partial pressure of oxygen or water vapor in the gas mixture changes during the measurements. Therefore, when studying the sensor sensitivity to reducing gases under light activation, additional efforts should be made to separate the “true” sensor response from the interfering factors.

## 6. Concluding Remarks

The analysis of the literature shows that the light activation of the sensor sensitivity of semiconductors is actively developing direction. The problems to be solved lie in the plane of both fundamental science and practical applications. Fundamental questions are related to the development of theories describing the effect of light radiation on the interaction of particles on the surface of semiconductors. The prospects for practical application are related to the possibility of reducing energy consumption when detecting gases.

The materials and approaches used to create light activated resistive sensors are quite diverse. The data obtained can be summarized as follows: (i) photoactivated sensors, as a rule, show the greatest sensitivity to oxidizing gases, while the sensor response to reducing gases is much less; (ii) a fruitful idea implemented for sensors with visible and UV light activation is the separation of photoexcited charge carriers between the two phases; (iii) the most promising approach to sensitize wide-gap metal oxides to visible light is the use of particles of narrow-gap semiconductors and semiconductor QDs.

Despite the large number of published articles, many questions concerning the light activation of the sensor sensitivity of semiconductors remain debatable. The disadvantage of many works is incomplete information about the parameters of the radiation used: the spectrum and power of light sources, the illuminance of the sensor layer, and the method of its determination. In this regard, it should be noted that the works on the develop of integrated miniature devices that combine the sensor layer and the light-emitting element in close to each other, which allows solving the problems of both reproducibility and the most efficient use of light energy [188,189,190,191]. Not all authors provide data on the long-term stability of sensitized materials containing reactive particles. Significant gaps remain in the understanding of the mechanism of sensory response formation. Nevertheless, the work on the light activation of the sensor sensitivity plays an important role in modern materials science due to its interdisciplinary nature and the prospects for practical application of the results.

## Figures and Tables

**Table 1:** Band gap (*E_g_*) values for metal oxides (*d*—for direct allow transitions, *i*—for indirect allow transitions, *f*—for direct forbidden transitions).

Metal Oxide	SnO_2_	ZnO	TiO_2_	In_2_O_3_	WO_3_
Crystal structure	rutile	wurzite	rutile	bixbyite	monoclinic
*E_g_*, eV	3.5 *^d^* [11]	3.4 *^d^* [12]	3.2 *^i^* [13]	3.75 *^d^*, 2.6 *^i,f^* [14]	3.5 *^d^*, 2.6–2.8 *^d,i^* [15]

**Table 2:** Sensor signal of wide-gap oxides in the detection of various gases when activated by ultraviolet and visible light. CBD—chemical bath deposition, RF—radio frequency; RT—room temperature; FA—formaldehyde, EtOH—ethanol, AC—acetone, LPG—Liquefied petroleum gas; NFs—nanofibers, NRs—nanorods, MSp—microspheres, NShs—nanosheets, NSp—nanosperes, MP—mesoporous.

No	Sensing Material	Synthesis Method	Detected Gas	Concentration, Ppm	Temperature, °C	Irradiation Parameters	Sensor Signal ^1^	Refs
1	2	3	4	5	6	7	8	9
1	SnO_2_	spray pyrolysis	AC	54	RT	Mercury lamp, >200 nm, 20 mW/cm^2^	1.8	[31]
3	nanocones SnO_2_	hydrothermal	H_2_	100	50	LED, 313 nm, 40W/m^2^	9.7	[35]
4	SnO_2_	RF sputtering	O_3_	5	RT	LED, 370 nm	1.3	[36]
5	In_2_O_3_	MOCVD	O_3_	0.5	RT	LED, 400 nm	1.6	[37]
6	MP In_2_O_3_	nanocasting	NO_2_	5	50	LED, 400 nm	7	[38]
7	In_2_O_3_	electrospinning	NO_2_	1	RT	LED, 400 nm	60	[39]
8	In_2_O_3_	sol-gel	NO_2_	8	RT	LED, 380 nm, 5 mW/cm^2^	180	[40]
9	In_2_O_3_ nanooctaedra	CVD	NO_2_	0.5	RT	LED, 325 nm, 400 μW	1.8	[41]
10	walnut-like In_2_O_3_	hydrolysis	NO_2_	2	RT	LED 365 nm, 1.2 mW/cm^2^	3.6	[42]
11	MP In_2_O_3_ NRs	hydrothermal	NO_2_	1	RT	365 nm, 6 W	20.9	[43]
12	ZnO hollow MSp	template synthesis	EtOH	100	80	LED 360 nm, 2 mW/cm^2^	11	[45]
13	ZnO NFs	electrospinning	FA	100	RT	LED 365 nm	12.61	[46]
14	ZnO NRs	hydrothermal	H_2_S	25	RT	LED 354 nm, 1.22 μW/cm^2^	3.55	[48]
15	ZnO NFs	electrospinning	EtOH	60	RT	Mercury lamp, 365 nm	1.75	[49]
16	WO_3_ NFs	electrospinning	AC	12.5	350	LED 365 nm, 2.024 mW/cm^2^	1.7	[56]
17	WO_3_/Au	RF sputtering	NO_2_	10	RT	LED 400 nm, 15 mW/cm^2^	~2.2	[57]
18	TiO_2_	Degussa P25	FA	100	RT	LED 365 nm, 36 W/m^2^	9385.5	[61]
19	TiO_2_	RF sputtering	NO_2_	100	RT	LED 365 nm	2.3	[63]
20	TiO_2_ NFs	electrospinning	H_2_	100	190	UV lamp, 300-400 nm, 3.25 mW/cm^2^	45	[64]
21	ZnO/Au NShs	sputtering	NO_2_	1	RT	365 nm, 1.2 mW/cm^2^	2.0525	[65]
22	ZnO NWs /Au	sputtering	EtOH	100	RT	254 mn, 4.1 mW/cm^2^	1.18	[66]
23	ZnO/Au	RF sputtering	H_2_	1000	250	365 nm	1.72	[67]
24	ZnO/Au NRs	thermal evaporation	O_3_	0.03	RT	LED 370 nm; 200 μW	~108	[68]
25	ZnO/Ag	CBD	NO_2_	5	RT	LED 365 nm, 8 mW/cm^2^	1.98	[70]
23	ZnO/g-C_3_N_4_	in situ precipitation	EtOH	104	RT	365 nm	4.26	[71]
24	SnO_2_/Pt clusters	RF sputtering	LPG	200	RT	UV lamp, 365 nm, 2 μW/cm^2^	4374.4	[72]
25	SnO_2_/Pd	wet-impregnation	NO_2_	5	30	LED 365 nm, 7 mW/cm^2^	1655	[74]
26	SnO_2_/rGO hollow NFs	electrospinning	NO_2_	3	RT	UV lamp, 365 nm, 97 mW/cm^2^	~2	[78]
27	ZnO/In_2_O_3_	coprecipitation	NO_2_	5	RT	LED 365 nm, 25 mW/cm^2^	3.21	[81]
28	ZnO/SnO_2_	ball milling	EtOH	10	250	LED 380 nm, 60 mW	10	[83]
29	ZnO/SnO_2_ NRs	CBD	NO_2_	0.5	20	LED, 380 nm	~1065	[84]
30	ZnO/SnO_2_	hydrothermal	O_3_	0.02	26	LED 325nm; 200 μW	8	[85]
31	ZnO/SnO_2_ hollow NSp	hydrothermal	FA	100	RT	LED 365 nm, 2 mW	~8	[86]
32	ZnO/SnO_2_ NFs	electrospinning	FA	50	RT	LED 365 nm	2.3	[88]
33	TiO_2_/SnO_2_	ALD	FA	0.6	RT	LED 365 nm, 10 mW/cm^2^	~5	[89]
34	SnO_2_/GaN NWs	MBE / RF sputtering	methanol	500	RT	Deut. lamp, 215-400 nm, 3.25 nW/cm^2^	~1.016	[91]

^1^ Sensor signal values are given (and recalculated, if needed) in accordance with formula (1). Since some values are extracted from graphical data, so some inaccuracy is acceptable. If in the article there were data for a set of sensors, the results are given for the best sensor.

**Table 3:** Sensor signal of sensitized wide-gap oxides in the detection of various gases when activated by visible light. CBD—chemical bath deposition; RT—room temperature; FA—formaldehyde, EtOH—ethanol, AC- acetone; NRs—nanorods, NWs—nanowires, MP—mesoporous.

No	Sensing Material	Synthesis Method	Detected Gas	Concentration, Ppm	Temperature, °C	Irradiation Parameters	Sensor Signal ^1^	Refs
1	2	3	4	5	6	7	8	9
1	SnO_2-x_	magnetron sputtering	EtOH	4.5	155	blue LED	0.286	[96]
2	WO_3_	commercial powder	NO_2_	0.16	RT	LED 580 nm, 340 mW/cm^2^	9.2	[98]
3	WO_3_ NFs	electrospinning	NO_2_	0.4	75	LED 430 nm, 770 μW/cm^2^	12.4	[99]
4	MP WO_3_	template synthesis	AC	100	25	475 nm, 40W/m^2^	7.5	[100]
5	WO_3_/PdO	hydrothermal/thermal	H_2_	40	RT	LED 480 nm, 0.15 W/cm^2^	9.02	[75]
6	ZnO	commercial powder	AC	900	25	visible LED	1.2	[101]
7	ZnO/In_2_O_3_	hydrothermal	FA	100	RT	monochromator, 460 nm, 0.213 mW/cm^2^	4.19	[102]
8	ZnO/In_2_O_3_	solvothermal	EtOH	100	260	Xe lamp, >420 nm	68.19	[82]
9	ZnO	ball milling	FA	100	RT	white LED (400–800 nm, 35.5 mW/cm^2^)	2.33	[103]
10	MP In_2_O_3_	nanocasting	O_3_	0.22	RT	LED 460 nm, light int. approx. 10 cd	120	[104]
11	V_2_O_5_ thin film	spray pyrolysis	AC	sat. vapour	RT	green laser, 200 mW/m^2^	3.53	[117]
12	Fe-doped ZnO (1%)	hydrothermal	FA	100	RT	laser 532 nm, 20 mW/cm^2^	2.87	[121]
13	Co-doped ZnO (1%)	coprecipitation	EtOH	18421	RT	monochromator, 630 nm	100	[122]
14	ZnO/porph. complex	dip casting	triethylamine	5500	RT	white LED,	~1.55	[136]
15	ZnO/RuN3	drop casting	CO	27894	RT	monochromator, 545 nm	1.5	[140]
16	In_2_O_3_/ Ru(II) complex	drop casting	NO_2_	2	RT	LED, 630 nm	100	[141]
17	ZnO/N719-dye	dip casting	NO_2_	1.25	RT	LED, 480 nm, 370 mW/cm^2^	1.43	[142]
18	SnO_2_/PI	impregnation	NO_2_	0.5	30	white LED, 400-700 nm, 3W	131.6	[144]
19	ZnO/Ag_2_S	cation exchange	EtOH	500	RT	laser 532 nm, 2 mW/cm^2^	45	[146]
20	CuO(4.17%)/ZnO	sol-gel	AC	500	30	Xe lamp, 420-780 nm	201.74	[147]
21	ZnO/CdS	CBD	FA	660	RT	Xe lamp, >450 nm cut-off filter	3.81	[148]
22	CdS/TiO_2_	SILAR	FA	100	RT	LED, 400–800 nm, 35.5 mW/cm^2^	2.54	[151]
23	CdS/ZnO	liquid plasma spray	NO_2_	1	RT	LED 510 nm, 50 mW/cm^2^	31.9	[153]
24	ZnO/CdS	spray pyrolysis	FA	10	29	400–800nm, 34.01mW/cm^2^	2.646	[153]
25	ZnO/CdSe nanoribbons	therm. decomposition	EtOH	25	160	Xe lamp, 12.18 mW	11	[154]
26	ZnO/CdS@ZnTe QDs	drop cast	NO_2_	1	RT	LED 535 nm, 20 mW/cm^2^	18	[160]
27	ZnO/CdSe QDs	drop cast	NO_2_	0.85	RT	LED 535 nm, 20 mW/cm^2^	20	[161]
28	ZnO/InP QDs	drop cast	NO_2_	1	RT	LED 535 nm, 20 mW/cm^2^	10.2	[164]
29	ZnO /PbS QDs	CBD	NO_2_	1	RT	LED 850 nm, 1 mW/cm^2^	1.24	[166]
30	ZnO/Au NWs	CVD / sputtering	C_2_H_2_	100	RT	laser 532 nm	~1.2	[169]
31	ZnO/Au NRs	sputtering	NH_3_	500	RT	> 400 nm, 60 mW/cm^2^	1.68	[170]
32	ZnO/Au	sputtering-annealing	EtOH	500	RT	mercury lamp, 600 mW/cm^2^	62	[172]

^1^ Sensor signal values are given (and recalculated, if needs) in accordance with formula (1). Since some values are extracted from graphical data, so some inaccuracy is acceptable. If in the article there were data for a set of sensors, the results are given for the best sensor.

## Data Availability

Not applicable.
